# Migration-related land use dynamics in increasingly hybrid peri-urban space: insights from two agricultural communities in Bolivia

**DOI:** 10.1007/s11111-018-0305-7

**Published:** 2018-10-29

**Authors:** Johanna Carolina Jokinen

**Affiliations:** Department of Social and Economic Geography, Uppsala University, Box 513, SE-751 20 Uppsala, Sweden

**Keywords:** Transnational labor migration, Agricultural change, Land use change, Peri-urban space, Latin America, Bolivia

## Abstract

This article investigates the impact of transnational labor migration on agriculture in urbanizing communities in Bolivia. Previous research shows that the characteristics of rural out-migration communities affect whether agricultural practices are intensified and improved. Using a mixed methods approach, two closely located peri-urban communities with distinct prerequisites for agricultural development are analyzed. This study shows weak migration-induced changes in agriculture and concludes that transnational migration does not necessarily accelerate an ongoing urbanization process. It shows that remittances function to maintain farming for subsistence and as a secondary livelihood activity. However, major investments in agricultural intensification are not attractive due to the communities’ proximity to the main cities. This article highlights the need for nuanced conceptualization when studying migration-driven agricultural change in hybrid peri-urban spaces.

## Resumen

Este artículo investiga el impacto de la migración laboral transnacional en la agricultura de comunidades en proceso de urbanización en Bolivia. Investigaciones anteriores muestran que las características de las comunidades rurales de donde provienen los migrantes inciden sobre la intensificación y mejora de las prácticas agrícolas. Utilizando un enfoque de métodos mixtos, se analizan dos comunidades periurbanas, geográficamente cercanas, y con prerrequisitos diferentes para el desarrollo agrícola. Este estudio muestra cambios débiles en la agricultura inducidos por la migración y concluye que la migración transnacional no necesariamente acelera el proceso creciente de urbanización. Muestra también que las remesas permiten mantener la agricultura como actividad secundaria para la subsistencia. Sin embargo, grandes inversiones en la intensificación agrícola no son atractivas, debido a la proximidad de las comunidades a las ciudades principales. Este artículo destaca la necesidad de una conceptualización matizada al momento de estudiar el cambio agrícola impulsado por la migración en espacios híbridos periurbanos.

## Introduction

**Fig. 1:**
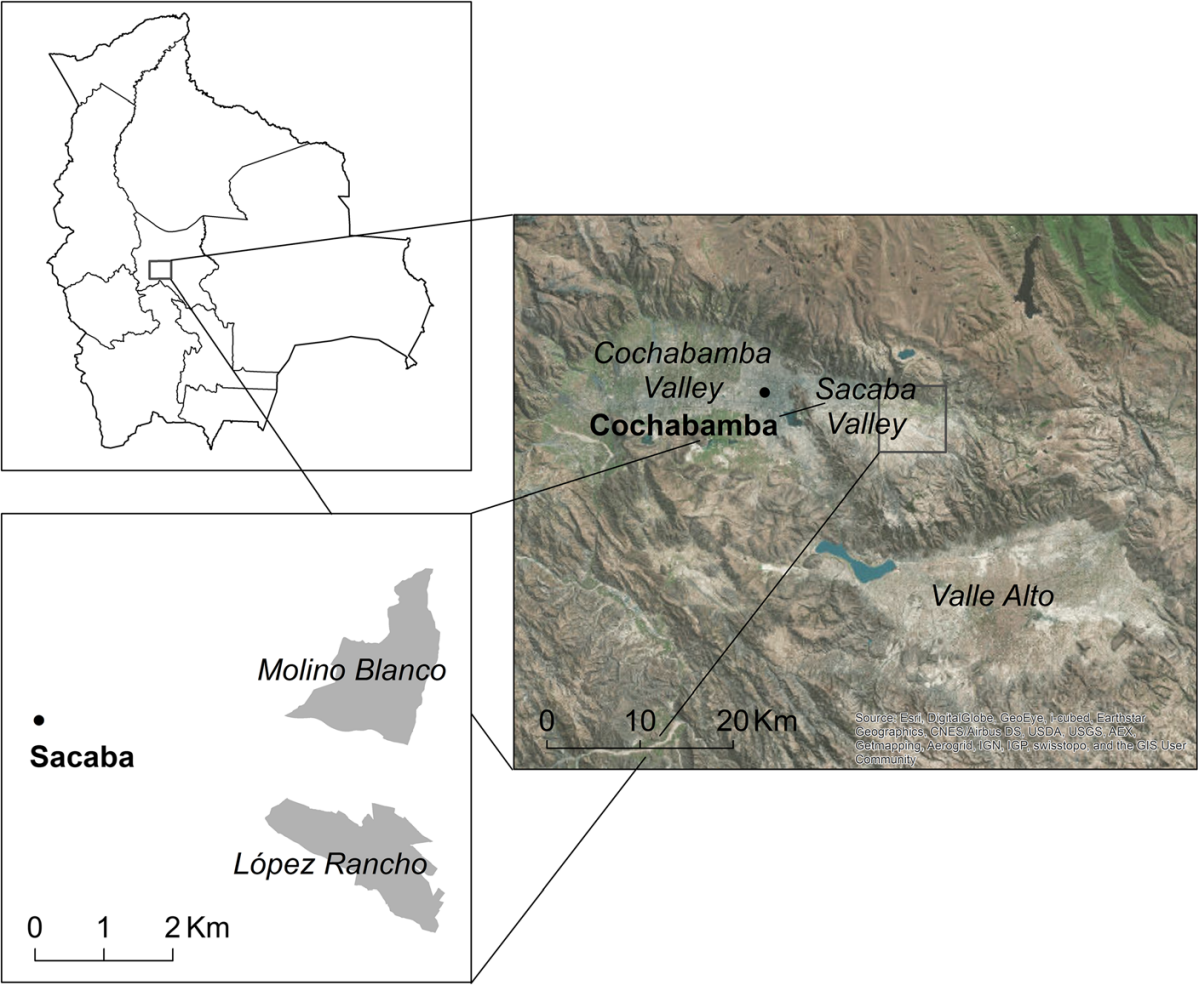
Location of López Rancho (65° 59′ 21 ′′ W, 17° 25′ 33 ′′ S) and Molino Blanco (65° 59′ 25′′ W, 17° 23′ 46 ′′ S) in relation to the three valleys in the department of Cochabamba. Source: Maps are originals by the author; basemap is provided by Esri, DigitalGlobe, GeoEye, i-cubed, Earthstar Geographics, CNES/Airbus DS, USDA, USGS, AEX, Getmapping, Aerogrid, IGN, IGP, swisstopo, and the GIS User Community

Throughout Latin America, agricultural workers are increasingly seeking new opportunities through transnational migration. At the same time, hybrid peri-urban land use combining farming with non-farm livelihoods has become an important component of food security in growing Latin American cities. This mixed methods research investigates how transnational labor migration is affecting agricultural practices and land use through an investigation of two peri-urban communities in the high-altitude, grain-producing region outside the city of Sacaba in Cochabamba, Bolivia (Fig. 1). Two major concerns motivate this investigation. First, the concern is that migration will cause agricultural households to either intensify or disintensify their farming practices. Second, particularly if agricultural livelihoods become less viable, the concern is that agricultural land will be converted to urban land and peri-urban households’ food security will be undermined.

Bolivia is a strategic site for exploring the relationship between agricultural practices and migration in peri-urban areas, where “peri-urban” refers to a transition zone between the rural-urban dichotomy that is “rural but not yet urban” (Lerner and Eakin 2011, p. 312). Although South America is the world’s most urbanized region, Bolivia remains one of the least urbanized countries within the region while simultaneously undergoing one of the continent’s highest rates of urbanization (Moreno et al. 2016). Bolivia has several rapidly growing metropolitan zones that include approximately 70% of the country’s urban population: Santa Cruz, La Paz, El Alto, and Cochabamba (Andersen 2002). Internal migration to the city of Cochabamba primarily originates from the departments of Potosí, Oruro, and La Paz of the *Altiplano*, the Bolivian high plateau. This migration drove the population of the metropolitan zone of Cochabamba to increase from 0.5 to 1.2 million between 1992 and 2009. Since the mid-twentieth century, Cochabamban people have been migrating transnationally, mostly to Argentina and the USA, while Spain, Italy, and Brazil became important destination countries in the early 2000s (Ledo 2012).

The Sacaban communities of López Rancho and Molino Blanco provide a novel context for investigating the effect of transnational migration on agricultural practices given their peri-urban location and distinct prerequisites for agricultural improvement. Several previous studies demonstrate the positive impacts of transnational migration on agricultural practices in rural Valle Alto, located outside the Central Valley of Cochabamba in Bolivia (see, e.g., Baby-Collin et al. 2008; de la Torre Ávila 2006; de la Torre Ávila and Alfaro Aramayo 2007; Strunk 2013; Yarnall and Price 2010). Less has been written about the agricultural communities that are in close proximity to the major cities of Cochabamba and Sacaba (see Ledo 2012; Umbarila et al. 2015). The agricultural landscape has already started to transform via a growing number of large, Western-style houses funded by remittances, reminiscent of the migration-driven development in Valle Alto. This study explores the role of transnational labor migration and remittances in creating a hybrid peri-urban space where urban neighborhoods are surrounded by agricultural fields of both subsistence and nontraditional commercial crops.

In the following section of this article, I review relevant theories and previous research on migration-related agricultural change. This review is followed by a section describing the study design, research methods, and data sources. The empirical results are presented in four subsections: (1) households’ involvement in agriculture and other livelihood activities in López Rancho and Molino Blanco; (2) agricultural land use with poor irrigation, weak local governance, and uncontrolled in-migration in López Rancho; (3) agricultural land use with abundant irrigation, efficient local governance, and controlled in-migration in Molino Blanco; and (4) the impacts of transnational migration on agricultural productivity in López Rancho and Molino Blanco. The concluding discussion addresses the theme of migration-induced agricultural change in the studied peri-urban communities. On the one hand, migration may result in labor loss, which can be compensated for by adopting labor-saving farming practices. On the other hand, monetary and social remittances may be key to making improvements in farming techniques.

## Impacts of migration-induced labor loss and remittances on agricultural land use

The new economics of labor migration theory proposes that migration is a household-level income diversification strategy to reduce economic risks (e.g., Massey et al. 1993; Taylor 1999). Household members’ seasonal, circular, and temporary migration minimizes the risk of employment loss and market failures (e.g., de Haan 1999; Ellis 1998; McCarthy et al. 2009). This study focuses on transnational migration, defined here as international migration in which migrants maintain their family ties across borders, for instance, through making regular visits, sending remittances, or having plans to return (see also Levitt and Jaworsky 2007).

While previous research acknowledges the importance of peri-urban agricultural communities as food providers for growing urban populations (Lerner and Eakin 2011; Tacoli 2003), it does not explicitly address how transnational migration affects agricultural land use in peri-urban out-migration communities. Whereas urbanization is often viewed as a threat to farming, the proximity of agricultural production to urban markets might actually be advantageous for peasant households (Lerner and Eakin 2011; Satterthwaite et al. 2010). Some policy analysts recommend the management of land use in peri-urban areas so that urban expansion does not compromise food production (Moreno et al. 2016).

Most previous studies on migration and agricultural change are based on an understanding of space in terms of a clear divide between the urban and the rural without considering how peripheral spaces surrounding cities often combine urban and rural characteristics (see also Dick and Schmidt-Kallert 2011; Lerner and Eakin 2011). Although researchers have analyzed the impact of transnational migration on agricultural land use and farming practices in rural villages, there is no consensus on whether smallholder agricultural systems are deteriorated, are reinforced, or mainly remain unchanged through migration and the monetary and social remittances received (see, e.g., Gray and Bilsborrow 2014). Moreover, it has been suggested that transnational migration and remittances have time- and place-specific effects that largely depend on the contextual characteristics of origin communities and their potential for agricultural improvement (e.g., Aguilar-Støen et al. 2016; Gray 2009; Jokisch 2002; see also Massey et al. 1994).

Globalization and migration are broadly observed to have led to agricultural abandonment and reforestation, particularly in mountainous regions and other marginal lands in Latin America (Aide and Grau 2004; Grau and Aide 2008). However, place-specific studies show that such a generalization represents a narrow understanding of the possible land use impacts of migration (e.g., Gray and Bilsborrow 2014; Radel and Schmook 2008). For instance, in a smallholder frontier of the Brazilian Amazon, migrants made up a surplus workforce; thus, their out-migration did not considerably affect agricultural land use (VanWey et al. 2012). In Guatemala, initial migration-induced deforestation due to investment in extensive farming practices was subsequently counterbalanced by forest recovery when economic and social remittances allowed the increasing participation of migrant households in nonagricultural work (Taylor et al. 2016). Moreover, research shows that migration and migrant remittances have led to the peri-urbanization of migrants’ origin communities through the expansion of residential areas at the expense of agricultural production (Jokisch 2002; see also Yarnall and Price 2010). Migration is also associated with agricultural extensification, i.e., decrease in agricultural inputs per land area, due to a diminished labor force. For instance, the transformation of cultivated land into grazing land, often in combination with remittance-financed land purchases, is a process of extensification (e.g., Black 1993; Preston et al. 1997; Sikor et al. 2009). This observation is consistent with the finding that migration-induced labor loss may result in land degradation (Harden 1996; Zimmerer 1993) due to insufficient maintenance of landesque capital, i.e., previous investments in soils and landscape infrastructure that improve the long-term productivity of farming land (Blaikie and Brookfield 1987; Erickson and Walker 2009; Widgren 2007).

Other research shows that migrants’ remittances are associated with greater investment in agriculture. Some studies have reported migration-induced agricultural intensification as remittances are used, for example, to introduce cash crops, to purchase fertilizers and pesticides, and to drill wells for irrigation purposes (de Haas 2006; Moran-Taylor and Taylor 2010). In favorable biophysical and structural contexts, remittances may, in fact, contribute to the commercialization of smallholder agriculture when they are used, for instance, to acquire more land and to compensate for labor losses through investment in livestock, paid labor, machinery, and irrigation infrastructure (Wouterse and Taylor 2008; Yarnall and Price 2010). Thus, remittances also contribute to preserving and creating anthropogenic landesque-capital landscapes (see Fisher and Feinman 2005) through “skills, technology, and labor that are directed towards improving infrastructure and enhancing soils on existing fields or new land that was previously unusable” (Erickson and Walker 2009, p. 234). Time is another factor influencing the relationship among migration-related labor loss, migrant remittances, and land use. Research in South Africa on transnational labor migration in the mining industry found decreased crop production shortly after the initial labor withdrawal from origin communities; however, over the long term, remittances were invested and ultimately resulted in greater agricultural productivity (Lucas 1987). Across different contexts, smallholder farmers are often able to cope with the negative effects of labor loss due to out-migration in the short term and benefit from remittances over a longer term (Gray 2009).

In addition to the negative and positive impacts of transnational migration on agricultural productivity, some studies show evidence of no impact. Smallholder farmers may choose not to invest remittances in agricultural inputs (Black 1993), as suggested by the fact that although rural families with migrant household members commonly have a higher income level than do non-migrant households, they do not necessarily have higher agricultural incomes (McCarthy et al. 2009) or more productive technology (Li and Tonts 2014). While agriculture is not abandoned, it often becomes a secondary income source (Black 1993; McCarthy et al. 2009; Preston et al. 1997), persisting as a culturally and symbolically important low-risk economic activity (Jokisch 2002; Li and Tonts 2014). Particularly in contexts in which there are poor biophysical and structural conditions for agricultural expansion and low profitability of semi-subsistence farming, investment in agriculture is not attractive (Jokisch 2002; McCarthy et al. 2009).

The contextual dimensions of migration-driven agricultural change have also been examined by considering migrants’ gender and ethnicity. In a study conducted in the Philippines, women’s transnational migration involved their withdrawal from households’ agricultural decision-making, leading to a shift from low-risk subsistence farming to a less sustainable cultivation of commercial crops, e.g., beans (McKay 2005). In contrast, men’s migration led to partial abandonment of the previously introduced cash crop cultivation of chili peppers in a Mexican community, while remittances were still invested in less labor-intensive maize production involving lower risks (Radel and Schmook 2008). In a comparison of the migration of indigenous Maya and nonindigenous Ladinos from Guatemala to the USA, Ladinos’ investment in cattle farming was promoted by structural factors based on the long-standing racial discrimination of the Maya. Consequently, because Maya migrants had difficulties purchasing land and cattle, they mostly invested their remittances in the production of food crops (Taylor et al. 2006).

In summary, empirical case studies provide mixed and in some cases contradictory evidence for how transnational labor migration impacts agricultural practices and land use in the rural out-migration communities of the Global South. Whereas previous research has mostly been focused on rural out-migration communities, I ask whether transnational migration contributes to the abandonment of agricultural livelihoods in peri-urban communities, a space where agricultural householders and urban in-migrants with nonagricultural livelihoods compete for resources.

## Study design, methods, and data sources

This study is designed as a qualitative analysis of two neighboring agricultural communities with contrasting conditions for agricultural development (Table 1). In the downstream community of López Rancho, although agricultural production is partly fed by the canal irrigation system, agriculture-based livelihoods have become less secure, as droughts are increasingly common and the water available for irrigation is often insufficient. The available water and land resources are poorly governed due to deficient local organization, which has been accelerating an urbanization process driven by the in-migration of those from even more precarious parts of Bolivia. Only a few kilometers to the north, in the upstream community of Molino Blanco, the situation is quite different. All available land is used for farming a variety of crops and is supported by abundant water resources from surrounding mountain reservoirs and the well-organized irrigation canal system. Access to water and land is efficiently governed based on the Andean ayllu, a community-level organization originating from pre-Columbian customary land tenure rules (Beaule 2016; Pape 2009). As a way to maintain agricultural production and preserve good socioeconomic conditions, in-migration to the community is strictly limited by locally approved legislation, in which *originarios*, the original community members, are privileged (see Pape 2009). These two peri-urban communities are agricultural zones with mixed land use and livelihoods due to community members’ non-farm employment in nearby urban zones. Transnational migration is occurring increasingly in both López Rancho and Molino Blanco.

**Table 1:** Main characteristics of López Rancho and Molino Blanco (Mair 2012; Marañon 2015; Umbarila et al. 2015)

Village characteristics	López Rancho	Molino Blanco
Number of households	400	235
Establishment year	1913 (Lava Lava vice-canton)	1885 (Molino Blanco hacienda)
Altitude	2850 masl	2800–2900 masl
Main crops	Potato, maize, wheat, onion, broad beans, peas, alfalfa, flowers, fruits	Maize, wheat, onion, peas, broad beans, potato, vegetables, tubers
Main migration destinations	1. Brazil, 2. Argentina, 3. Chile	1. Spain, 2. Chile, 3. Argentina
Migrant households	5.6%	7.5%
Agriculture as main occupation	11.5%	10.2%
Good water quality	36.0%	68.0%
Access to bathroom	81.0%	96.0%

This investigation is based on interviews, observations, remotely sensed data, and focus groups. I conducted 44 interviews in 2013 and 2014, of which 25 were in López Rancho^1^ and 19 in Molino Blanco (Table 2).^2^ While I mainly interviewed smallholders to obtain diverse information on their experiences (see also Dunn 2005; Dwyer and Limb 2001), a few key informants with a broader view of the local agriculture were included in this study. These key informants also reside and have agricultural activities in the same communities. All the interviewees work at least part-time in agriculture, mostly on privately owned land. Both migrant and non-migrant households were included in this study.

**Table 2:** Interviewed farmers and their households’ experiences of migration in López Rancho and in Molino Blanco

Informant characteristics	López Rancho	Molino Blanco
Number of informants	25	19
Age (mean, standard deviation, range)	53.0, 19.6, 65	52.9, 15.7, 57.0
Male	44.0%	52.6%
Female	56.0%	47.4%
Non-migrant households	40.0%	57.9%
Migrant households with one migrant	20.0%	21.1%
Migrant households with several migrants	40.0%	21.1%
Migrant households migrating to one destination country	40.0%	31.6%
Migrant households migrating to several destination countries	20.0%	10.5%
Migrant households migrating to Spain	32.0%	21.1%
Migrant households migrating to Argentina	40.0%	10.5%
Migrant households migrating to Chile	0.0%	10.5%
Migrant households migrating to Italy	8.0%	5.3%
Migrant households migrating to the USA	8.0%	0.0%
Migrant households migrating to Israel	0.0%	5.3%

The research project was approved by locally elected community leaders, and the informant recruitment was facilitated by research assistants from a local university. The interviews were often conducted in close proximity to the informants’ homes to be able to understand and better observe their everyday life and living environment. Alternatively, the informants were interviewed while working on agricultural fields or pastoral lands. These occasions familiarized me with the farming techniques used and the challenges involved (see also Kearns 2005). Some interviews were complemented by performing field walks on the informants’ farmlands located in different parts of the communities and at varying altitudes. The interviews were mostly conducted in Spanish. Nonetheless, the informants were given the option to communicate in Quechua if doing so was needed to provide more accurate descriptions. As all the interviews were transcribed by my research assistants, the parts in Quechua were translated into Spanish during the transcription process. The interview data were analyzed by first organizing them thematically and by writing down my own comments and interpretations; then, the thematically important parts were arranged in comprehensive tables to present general patterns.

I also analyzed land use changes by using remotely sensed data. Historical aerial photographs from 1962, 1972, and 1983 were provided by the Military Geographical Institute and facilitated by the Bolivian Air Force. These images were visually compared with Google Earth® high-resolution satellite imagery from the 2000s. The purpose of this analysis was to identify major changes in agricultural land use, serving as a complementary data source. In addition, individual agricultural fields were digitized in ArcGIS to compare how their size changed between 1983 and 2013. The changes in total area of cultivated land were identified to understand the continuity of agricultural practices, and land fragmentation was analyzed by calculating changes in the mean area of individual fields.

After the interview material was collected, I organized a focus group in each community in 2014. During these focus groups, a few key informants together with smallholders provided more detailed and systematic information on the primary obstacles to agricultural activities, the characteristics of the cultivated crops, and changes in land use. Participatory exercises were used to reveal how the informants rank and describe the primary problems of agricultural development and how they value different crops by differentiating, for instance, among nutritional value, profitability, and intensity of labor (see Mikkelsen 2008). I also discussed the changing land use with the participants by presenting the historical aerial photographs from different years. Subsequently, the study results were presented in a seminar at the local university in 2015 and in a separate focus group in each community in 2016. These focus groups and the seminar functioned as a technique to review and triangulate the information that I had previously collected through interviews, observations, and land use analysis (see also Valentine 2001).

## Migration and agriculture in peri-urban communities of López Rancho and Molino Blanco

### Agriculture and other livelihood activities in López Rancho and Molino Blanco

During colonial times, wheat, barley, and maize were cultivated in great quantities in López Rancho and Molino Blanco to meet the demand for grains in the *Altiplano*. Several varieties of potatoes, broad beans, and alfalfa were also produced to a lesser extent (Jackson 1989). In addition to these traditional crops, study participants of both communities report incorporating peas, onions, and cut flowers into their farming practices. Onion cultivation was introduced in the 1990s, and cut flowers have been produced since the early 2000s. The products of cash-crop farming are sold in the markets of Sacaba and Cochabamba. Additional vegetables, quinoa, and fruits are produced in home gardens, mostly for subsistence. It is also common for families to have a few milk cows, sheep, chickens, rabbits, and guinea pigs, chiefly for household use and for occasional sales.

**Fig. 2:**
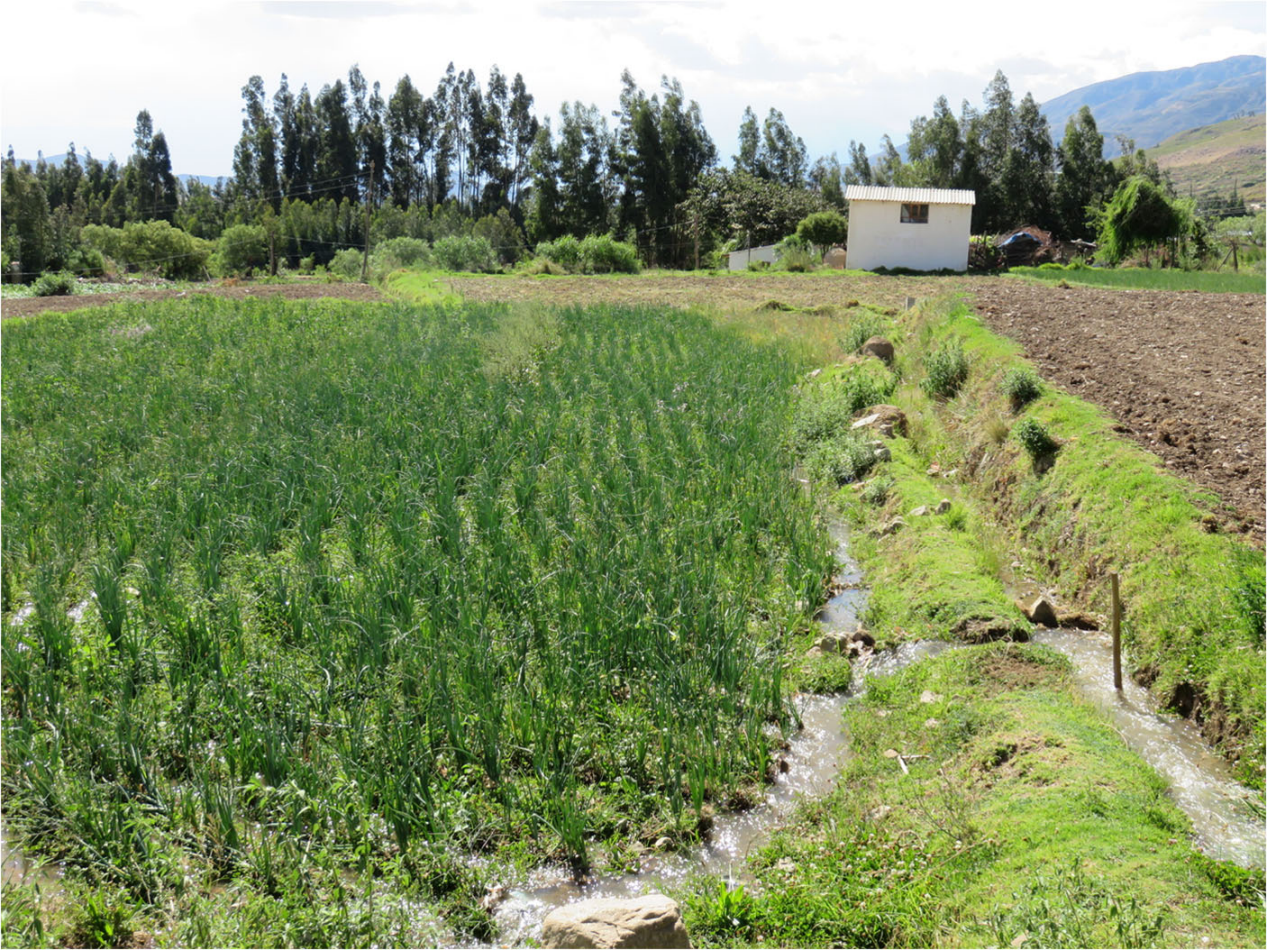
Surface irrigation by inundation in Molino Blanco

**Fig. 3:**
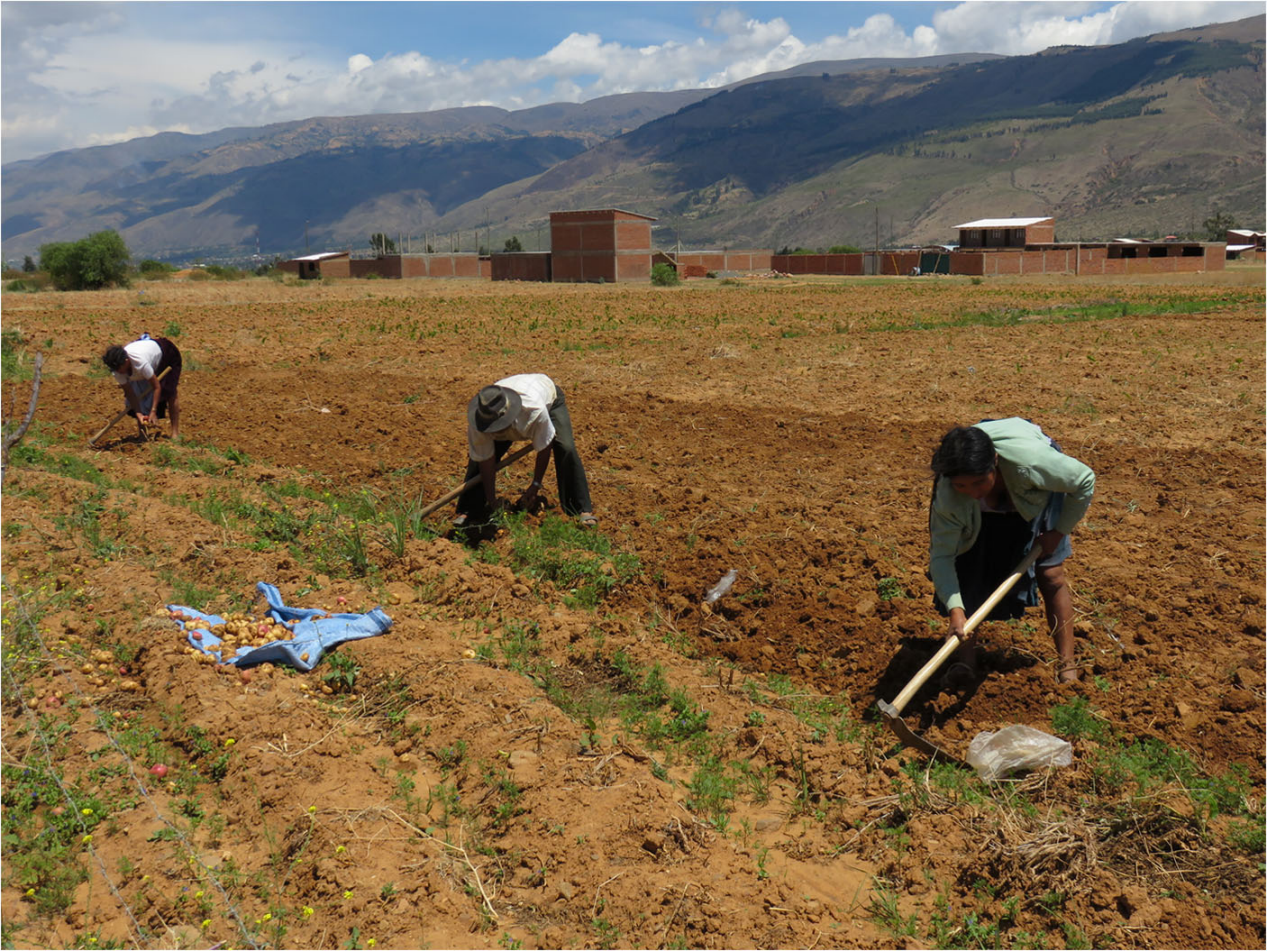
Use of manual techniques for potato harvesting in López Rancho

Irrigation and heavy pesticide use are necessary for most of the crops, particularly for the modern crops. In irrigated parts of López Rancho and in Molino Blanco, surface irrigation is conducted by inundating a field; then, the water is allowed to drain to the next field, often situated at a lower elevation (Fig. 2). While tractors are sometimes used for plowing and sowing instead of or in combination with oxen, harvesting is always performed manually in both communities (Fig. 3). Manual techniques are also commonly used to remove weeds; to *aporcar*, to put soil on the roots when the plants are still small; and to *cavar*, to dig small parallel channels between the plants to enable surface irrigation. *Cavar* is performed to prevent plants from being displaced by water. Since these channels are not always dug in an effective manner, this irrigation technique has contributed to soil erosion as the fertile topsoil layer is washed away. To address this problem, there are plans to introduce sprinkler irrigation.

Although both López Rancho and Molino Blanco are still classified as rural by the local administrative division, the prevailing agricultural activities are increasingly considered by the villagers to be an additional economic activity along with off-farm/non-farm employment and transnational labor migration. While a few of the households only have members working in agriculture and no other income sources, most of them have household members who have or have had other employment in Sacaba, in other locations in Bolivia, or abroad. For instance, one parent—often the father—may have other employment, while the spouse and some of the children perform most of the farming activities. Some households also have a small business at home, such as a small corner store or a *chichería* (a bar/brewery selling *chicha*, a fermented maize beverage that is consumed in large quantities in rural and peri-urban areas of Bolivia). Both in López Rancho and Molino Blanco, there is also an ongoing process of minifundization, i.e., land fragmentation because of inheritance. Most of the agricultural households own only 1.5 ha land or less, which makes it difficult to engage in farming as the main economic activity (Umbarila et al. 2015).

In López Rancho and Molino Blanco, typical off-farm employment includes working on nearby chicken farms and day labor for other farmers, such as plowing with oxen. The most common non-farm occupations include working as a taxi driver, bricklayer, and store clerk. Non-farm working opportunities, including transnational labor migration, are sought to supplement farm income, which can be an unstable income source and extremely demanding. “I was working as a cook in Spain almost for 10 years and I wanted to stay there as it is hard to work here; you need to irrigate, you need to *aporcar*” (Ana, 39 years old, Molino Blanco). Moreover, according to several study participants, the lack of local non-farm employment opportunities drives their household members to migrate transnationally. “If you do not go abroad, there is not much to do here in the countryside; it is very difficult” (Esteban, 30 years old, López Rancho/Lava Lava Alta). “My children are working in Argentina as there is no work here” (Miranda, 59 years old, López Rancho).

Whereas the villagers of López Rancho and Molino Blanco have primarily been migrating to Argentina and Spain, some residents have also moved to Chile, Italy, the USA, and Israel. Both men and women migrate, but in this study, I do not particularly investigate the gender aspect of migration and migration-related agricultural change. While men mostly work as bricklayers abroad, women are often employed in elderly care and cleaning occupations in Spain and as vegetable sellers and seamstresses in Argentina. Although the migrants originate from agricultural households, they do not normally engage in agricultural employment abroad.

Both in López Rancho and Molino Blanco, it is common for children to study and/or aspire to other forms of employment instead of continuing with farming, as presented in the following quotations. “I only work with farming as my children do not want to work in agriculture, brute work they tell me, and of course, it is brute work; they do not want to live in this way” (Julio, 80 years old, López Rancho). “No, my children do not want to continue with farming; they only grab their books and they have hardly been doing any agricultural work during this holiday. They only helped me a bit, to plant onions, but nothing else” (Teodoro, 50 years, Molino Blanco). In other interviewed households of López Rancho and Molino Blanco, however, some children and young adults are still interested in farming at least as a part-time activity. “I do not have other plans than continuing with agriculture. I would like to own land, but I do not have enough money to buy it” (Jennifer, 30 years old, López Rancho). “My son works as a bricklayer, but he also grows potatoes and peas for subsistence” (Aurelio, 62 years old, Molino Blanco). “My four sons have been studying; one of them is working as a soldier in Santa Cruz, and another one is an electrician, and my three daughters who have not been studying are continuing with agricultural activities” (Antonia, 72 years old, Molino Blanco).

While starting a small business or out-migrating transnationally is not always possible due to a lack of economic resources, there might also be a fear of losing everything due to failing to repay a bank loan. Hence, as also shown in the quotation below, farming can be viewed as the only available and feasible economic activity, especially by those of the elder generation, even though their children, often obtaining a higher educational level, might prefer other forms of employment.

I was thinking about taking a bank loan and starting a business, but it is also difficult, as you might go bankrupt and then, the bank would take away your house. So, I will continue working with agriculture. As my children have other employment, they can pay for their clothing and groceries. So, farming is only to make my living (Flora, 46 years old, López Rancho).

However, agriculture is considered by some villagers to be a traditional livelihood that cannot be abandoned, even though households have alternative sources of income. For instance, one informant with employment as a bricklayer shared that his father still insists on continuing to farm at the age of 71 even though the household can support itself by relying only on non-farm income. To conclude, agriculture remains an important livelihood activity in López Rancho and Molino Blanco despite the emerging urbanization process and households’ attempts to seek out other sources of income.

### López Rancho

López Rancho is a community in which there is poor irrigation for agricultural land, weak governance of community resources, and uncontrolled in-migration. There are 400 affiliated households in López Rancho, and in general, based on the interviews and observations, the villagers’ socioeconomic status is lower than in Molino Blanco. For instance, the inhabitants have lower access to bathrooms and safe drinking water than in Molino Blanco (Marañon 2015). While there is still land available to expand farming activities in López Rancho, the interviews and focus group exercises showed that the insufficient irrigation water and the poor irrigation infrastructure are the major obstacles for agricultural development.

There is inadequate local water management in López Rancho as the irrigation water is distributed by *Asociación de Regantes de Apaka Punta* (ARAP), an irrigation association governed by Molino Blanco and other upstream communities (see also Marañon 2015). López Rancho entered into the association after its foundation, and the community is only connected to the reservoir of Achocalla. Although they are normally allowed 14 water turns per year, the number of turns is minimized in the case of drought. While there has been an irrigation system since the early 2000s in López Rancho, it covers only a part of the community. In addition to the water distributed by ARAP, there is a communal drilled well, but the amount of water farmers are allowed to extract is limited. “We would need at least some three or four drilled wells to be able to irrigate all the fields; we could cultivate everything as there is land available here, but there is lack of water” (Miguel, 58 years old, López Rancho). Even if wheat and barley can be cultivated without irrigation, rainfall allows only one harvest per year. If there is not enough precipitation at the beginning of the rainy season, it may not be possible to sow at all. To allow storage of irrigation water and to decrease their dependency on precipitation and ARAP, community members are planning to build another reservoir by applying for financial assistance from the municipal government. However, the reservoir construction plans have been delayed for several years, which also indicates the villagers’ insufficient organization.

**Fig. 4:**
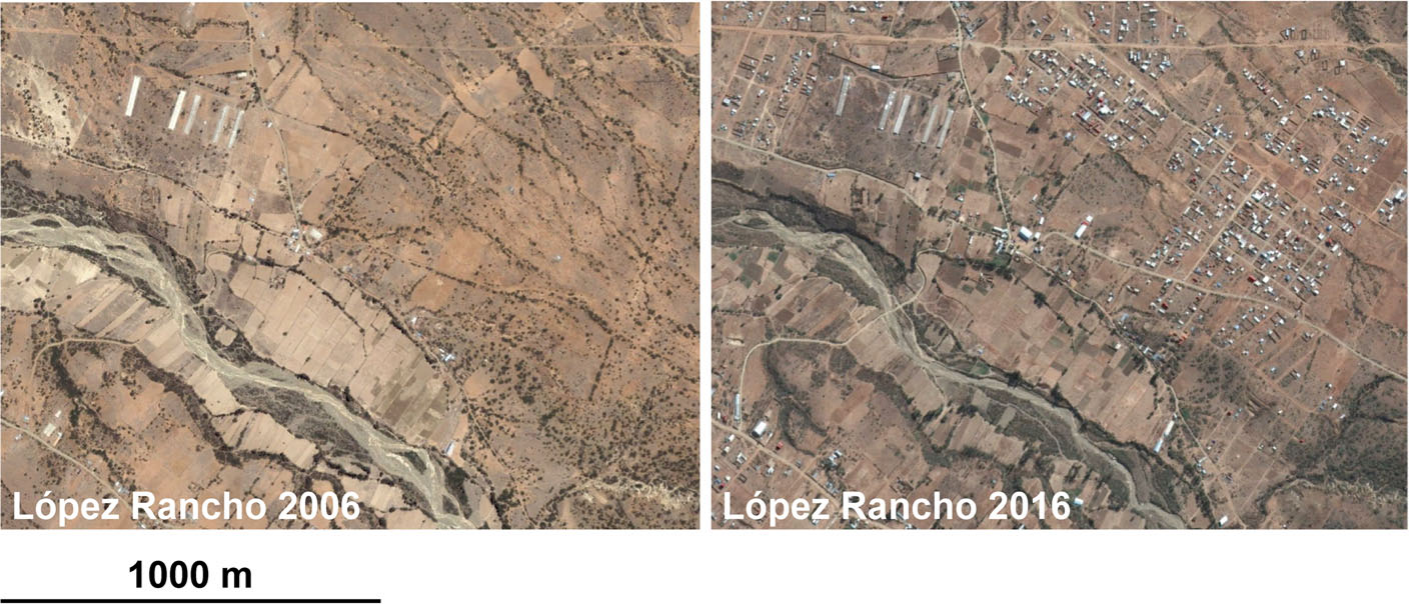
Two Google Earth® images of López Rancho from November 2006 and July 2016 showing that the urbanization by in-migrants mostly takes place in zones that have not been previously used for cultivation. Source: Google, DigitalGlobe

In López Rancho, internal in-migration has been rapidly growing since 2010/2011. This urbanization process is evident when comparing the satellite images from 2006 to 2016 (Fig. 4), and the community is currently divided between “the peasants” and “the urban dwellers,” the latter often originating from poverty-stricken rural areas of the *Altiplano*.

They [internal in-migrants] have been moving here since 8-10 years ago, mostly from Potosí, Sucre, El Alto, and Oruro. They do not participate in agricultural activities. There is no change in our agricultural land use yet because of these in-migrants, “the urban dwellers” (Miguel, 58 years old, López Rancho).

Due to this recent division and the groups’ different interests, it is difficult to agree on any local legislation governing the available water and land resources. Because of the lack of regulation, the urbanization process is accelerating further and there have been problems of illegal acquisition of uncultivated land for housing purposes. The study participants also admit that agricultural households of López Rancho occasionally need to sell their farming land for housing because of the diminished production in recent years.

**Fig. 5:**
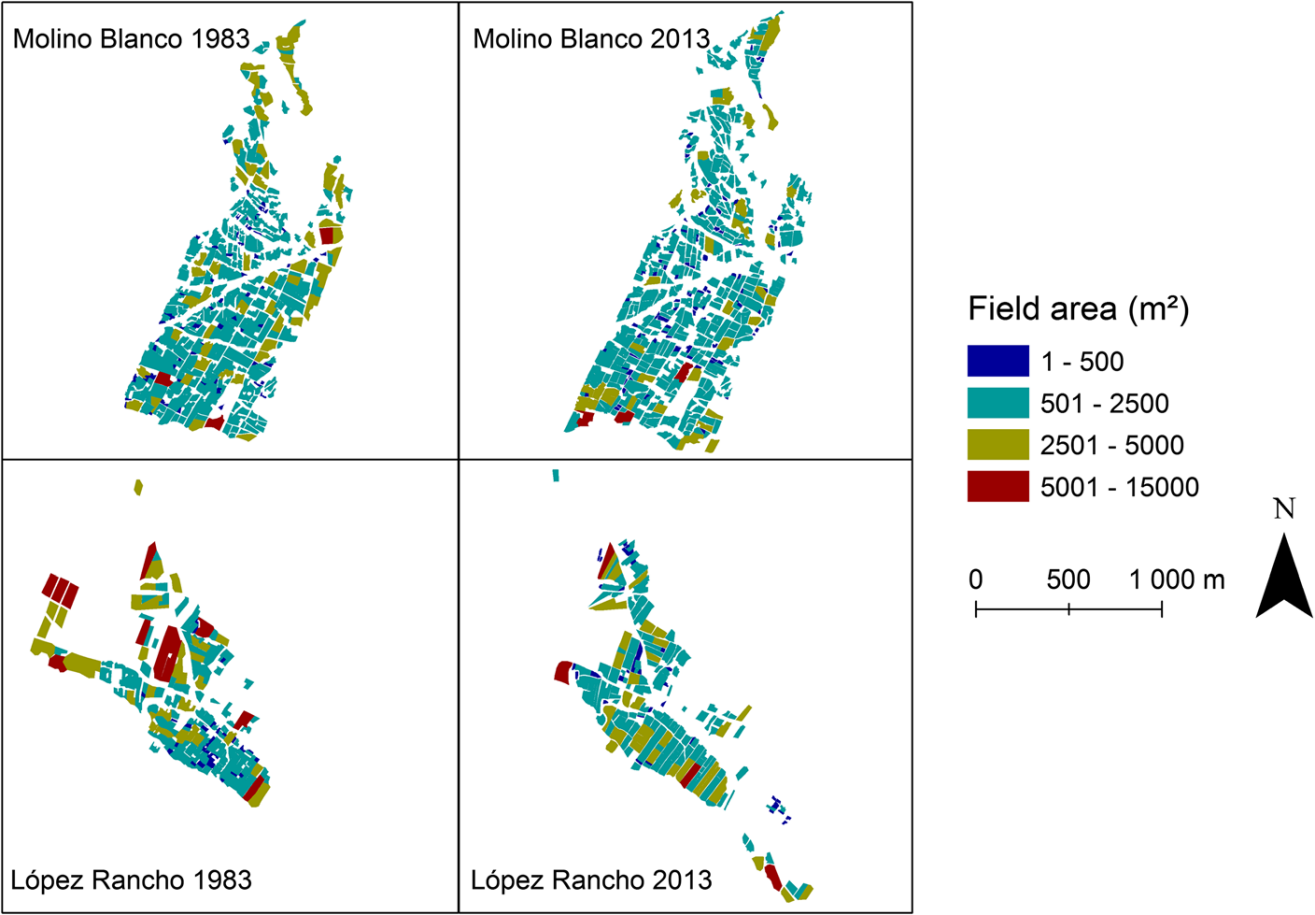
Changes in the area of individual cultivated fields in Molino Blanco and López Rancho from July 1983 to November 2013

Based on the output of remotely sensed data, to date, the recent urbanization process has had a somewhat limited impact on the total extent of agricultural land in López Rancho. While the mean area of individual fields has decreased by 15% due to land fragmentation, there was only a 3% decrease in the total field area from 1983 to 2013 (Table 3). Hence, urbanization has not yet resulted in the abandonment of agricultural land. The impact has been limited as most in-migrants reside in areas that have never been used for cultivation, i.e., abandoned pastoral land, cleared thicket/woodland, or previously unexploited land with unfavorable soil quality for farming. However, it is important to note that some fields are not productive anymore, mainly due to climatic stress and lack of irrigation, whereas some previously tree-covered areas have been converted into new fields for rainfed cereal cultivation (Fig. 5). Nonetheless, if the in-migration continues at a similar pace in the future, it will eventually become necessary to exploit agricultural land to a greater extent. Without legislation governing the transformation of rural land into urban land, it is challenging to stop land sales.

**Table 3:** Changes in characteristics of cultivated fields in López Rancho and Molino Blanco from July 1983 to November 2013

Field characteristics	Lopez Rancho			Molino Blanco		
	1983	2013	Change	1983	2013	Change
Number of fields	247	274	10%	521	628	17%
Field area, mean (m^2^)	1634.5	1427.5	− 15%	1400.1	1185.4	− 18%
Total field area (ha)	40.4	39.1	− 3%	72.9	74.4	2%
Field perimeter, mean (m)	170.0	159.7	− 6%	163.2	148.5	− 10%

### Molino Blanco

Molino Blanco is a community in which there are abundant irrigation water resources for agricultural land, efficient governance of community resources, and strictly limited in-migration. Molino Blanco consists of 235 affiliated households, and in general, the villagers have higher socioeconomic status than that of López Rancho residents. Whereas shortage and fragmentation of cultivable land threaten agricultural development in Molino Blanco, the year-round access to irrigation enables intensive production despite small field sizes (see also Umbarila et al. 2015).

Since colonial times, the agricultural fields of Molino Blanco have been irrigated through an advanced canal system that utilizes water originating from nearby mountain reservoirs. In 1998, the canal system was improved and Achocalla, the principal reservoir, was extended to accommodate more water. ARAP, the well-organized irrigation association governing water resources, was established the same year. There are 27 member communities in the association, and these syndicates receive water from different reservoirs. As Molino Blanco is one of the main shareholders and a founding member, the syndicate is connected to several water reservoirs. The local irrigation syndicate of Molino Blanco distributes water according to the size of community members’ agricultural land, and the associated farmers pay a monthly fee for their water use. Although the farmers claim that precipitation has been decreasing and temperatures have been increasing over the course of recent years, there is no lack of water due to the irrigation system in the community, as described below.

We are planting here in Molino Blanco [this year], but it is much worse in López Rancho and other [downstream] communities in the southern sector; they do not have any water [due to the drought]. There is always a bit more water in Molino Blanco than in other communities; it is our turn almost every week [even this year] (Claudio, 55 years old, Molino Blanco).

Hence, it is possible to harvest potatoes and flowers twice per year in Molino Blanco.

In comparison to López Rancho, the process of internal in-migration is strictly limited in Molino Blanco. As the community members form a more unified group of peasants, it has been possible to reach a consensus on local policies governing the water and land resources. Land sales are controlled by high affiliation fees that new community members are required to pay to become enrolled in the community and to get access to basic services. Thus, in-migrants from underprivileged regions of Bolivia cannot afford to live in Molino Blanco.

Becoming affiliated in the neighboring communities is cheap, BOB 500 [USD ~72], whereas it costs BOB 5,000 [USD ~722] here [in Molino Blanco]. And the fee will be raised to BOB 15,000 [USD ~2,165]. For inheritors of the villagers, it is BOB 500 [USD ~72] (Eduardo, 45 years old, Molino Blanco).

As stated above, the affiliation fee for families who are not inheritors of the original villagers is ten times the affiliation fees in other nearby communities, including López Rancho. In addition, community members are planning to triple the fee.

Aerial photographs and high-resolution satellite images of agricultural fields show that the total area of cultivated land in Molino Blanco increased by 2% from 1983 to 2013. The mean area of individual fields decreased by 18% and was 242.1 m^2^ smaller than in López Rancho in 2013 (Table 3). Although the overall extent of farming land has not been changing in Molino Blanco, the fragmentation of agricultural land through inheritance is experienced by several villagers as an important element ultimately causing the abandonment of agricultural activities. The dilemma is described in the following quotations. “We are dividing [land] because of hereditary succession, and later, we will not have land available for agriculture anymore” (Agustina, 51 years old, Molino Blanco).

In my opinion, inheritance is the most severe problem for the continuation of agricultural production in Molino Blanco because the land is distributed and, hence, it is not possible to continue working with farming anymore. Instead, you need to use the land to build a house for your family, and this factor is leading to the disappearance of agriculture (Pablo, 66 years old, Molino Blanco).

In addition to the house construction on inherited land, some informants mentioned that the community’s rural image is starting to be hampered because of occasional land sales for housing purposes.

The villagers want Molino Blanco to be a rural place, but they also sell land for house construction. For instance, they sell land to outsiders when they get sick if there are no other villagers who would like to buy. They are building houses down there; I think it is a lawyer [who bought the land] (Ana, 39 years old, Molino Blanco).

Returning migrants from Spain and other countries have bought land here to build their house in this village. I have asked why they buy land here, and they tell me, “It is a paradise here.” But, if we fill it up with cement, it is not going to remain a paradise (Pablo, 66 years old, Molino Blanco).

Accordingly, as demonstrated in the quotations above, land for housing purposes is increasingly bought by families with access to economic resources. Due to these families’ higher socioeconomic status, it is clearly not possible to thwart their intention to purchase land by requiring high affiliation fees for community membership. Nevertheless, as opposed to López Rancho, urbanization through in-migration is still heavily controlled in Molino Blanco, and some of the new community members also practice farming.

### Transnational migration as a driver of agricultural change?

Despite the loss of labor due to transnational migration, most families continue to farm. Both in López Rancho and Molino Blanco, several study participants mention that it has become difficult to hire laborers, as many working-age residents have been out-migrating or subsisting on non-farm employment. “There is no manpower as some people have become taxi drivers, some people have moved to other regions, and then other people have traveled to Santa Cruz or also abroad, so there are no people to work [in agriculture]” (Luis 45 years old, Molino Blanco). Instead of hiring other villagers, the farmers often need to search for laborers originating from other communities. “We need to be looking for workers; we normally go to the main road to bring them” (Flora, 46 years old, López Rancho). If the households cannot find any available laborers, they do agricultural work themselves or extensify land use. To compensate for the labor shortages when working-age household members are abroad, families will cultivate only part of their land or switch to alfalfa cultivation for livestock. The following quotation shows how a migrant household adjusted its workload.

We also cultivated when I was working in Spain for a period of almost 10 years but not as much as before. During that period, we only cultivated alfalfa for the cattle, as it is easier to take care of. You only need to irrigate, and you just leave it to grow for eight years or more. However, it does not give you any money; the cows eat it all. My mother and my sister worked with that when my husband and I were in Spain (Ana, 39 years old, Molino Blanco).

While most of the migrant households receive monetary remittances regularly or sporadically, the money is mainly used for everyday consumption and to finance studies, house construction, and the expenses of left-behind children. However, as demonstrated below, several study participants report occasionally using remittances to purchase seeds, pesticides, fertilizers, irrigation water, and services of tractor or oxen-driven plowing or to hire agricultural labor for certain tasks, such as planting and harvesting. “We buy seeds [with remittances, in addition to having been building houses, buying small plots of land, and saving a part of them]” (Elias, 63 years old, López Rancho). “We have also invested remittances in agriculture [in addition to buying a house in the city of Sacaba], as you need quite a lot of capital to hire tractors and [to hire workers] for harvesting” (Ana, 39 years old, Molino Blanco).

In fact, several study participants admit that it is not profitable to invest quantities of remittances in farming because of insufficient irrigation water in López Rancho and land shortage in Molino Blanco. “There is [cultivable] land available here that we would like to buy [with remittances], but we cannot because we do not have water” (Miguel, 58 years old, López Rancho). “Most of the used farming techniques are still manual, and it is only with certain crops, like wheat, that we use a combination of tractor and manual labor” (Luis, 45 years old, Molino Blanco). “Because of small plot sizes, it is not even possible to industrialize our agricultural production” (Pablo, 66 years old, Molino Blanco). As demonstrated by these quotations, there is very little evidence that remittances have resulted in higher agricultural productivity in López Rancho and Molino Blanco through investments in farming land, new methods, and equipment. Thus, non-migrant households and migrant households normally use the same traditional farming methods, even if the latter often have better economic possibilities to improve agricultural practices. Nevertheless, remittances have contributed to a minor increase in tractor use instead of oxen. It is more expensive to hire a tractor by the hour but as seen in the following quotation, it often turns out to be a more time and cost efficient to use such a plowing service than to use oxen. “When using oxen, the plowman drinks *chicha*, and once he’s drunk, he does not want to work anymore. It is therefore faster to plow with a tractor, as tractor drivers drink only soda” (Faustino, 80 years old, López Rancho).

A few migrant households have used remittances to buy agricultural land in nearby communities and in the department of Santa Cruz. “My brother [who was working as a construction worker in Spain] has bought two pieces of [agricultural] land in Santa Cruz” (Flora, 46 years old, López Rancho). “My mother [who is employed as an elderly care worker in Spain] is thinking of buying a farm in Santa Cruz to raise cattle and to grow fruits” (Judit, 19 years old, López Rancho/Lava Lava Alta). As shown by these quotations, migrant households with access to remittances are also looking for possibilities to invest in farming land in other locations in Bolivia. In contrast to both communities studied here, there is plenty of fertile farming land available in Santa Cruz, enabling a profitable livelihood, for instance, through raising cattle.

Although migration-driven changes to agriculture are generally limited to extensification in both López Rancho and Molino Blanco, there are a few exceptions. For instance, a couple residing in López Rancho worked for 6 years in Spain and used remittances to introduce cut flower cultivation in greenhouses.

Cut flower production is economically much better than other traditionally cultivated crops, but it requires a lot of investment. First, you need to have a drilled well since, without water, it is not possible to do anything. Then, if you have a greenhouse, you can produce anything. Without a greenhouse, you just seed in vain, as the moisture and the heat paralyze your plants. If we had not gone to Spain, it would have been completely impossible for us to achieve this kind of cut flower production (Esteban, 30 years old, López Rancho/Lava Lava Alta).

As the quotation above shows, monetary remittances enabled investment in cash-intensive agricultural production that is otherwise not common in Sacaba. Although cut flowers are cultivated in López Rancho and Molino Blanco, the other famers do not produce them in such a commercialized manner in greenhouses. In addition to monetary remittances, out-migration in some cases increases knowledge of new kinds of crops through social remittances, as presented in the following quotation.

I also have green beans [growing in my garden]; they sent me the seeds from Spain. One of my sons lives there. And my wife’s daughter lives in Rome in Italy, and her sister lives in Milan, and they brought us carrot seeds in a small envelope, and white onion seeds as well (Pablo, 66 years old, Molino Blanco).

These two examples provide an exception to the overall result of minimal migration-related changes in farming practices and land use. As opposed to most migrant families in López Rancho and Molino Blanco, these two households have family members who have been studying agronomy or who otherwise have detailed knowledge of different farming techniques, including their advantages and disadvantages.

## Concluding discussion

The findings of this study do not support previous investigations that suggest that labor migration results in the abandonment of agricultural land (see Aide and Grau 2004; Grau and Aide 2008) and its conversion to urban uses (see, e.g., Jokisch 2002; Yarnall and Price 2010). Even if out-migration and the increase in off-farm/non-farm employment opportunities have led to a decline in the available agricultural labor force in López Rancho and Molino Blanco, this labor loss is often temporarily compensated for by applying labor-saving practices, for instance, by cultivating only certain fields or by transitioning to crops that require less work. However, in contrast to the findings of previous research showing “remittance investments into cattle and pasture expansion” (Davis and Lopez-Carr 2014, p. 328; see also Taylor et al. 2006), land shortages in these communities preclude such extensification of agricultural activities. Due to their peri-urban location, there is no land available to switch to cattle rearing as a labor-saving adaptation. Nonetheless, a few households have extensified their farming by purchasing pastoral land in Santa Cruz.

Nor do the results of this investigation show migration-driven intensification and commercialization of agriculture, as reported in other studies (see, e.g., de Haas 2006; Moran-Taylor and Taylor 2010; Wouterse and Taylor 2008; Yarnall and Price 2010). In general, there are only a few observable differences between migrant and non-migrant households regarding their agricultural production (see also McCarthy et al. 2009) and farming techniques (see also Li and Tonts 2014). Although remittances are occasionally invested in agricultural inputs in López Rancho and Molino Blanco, for the most part, there are only minor changes in the crops cultivated and the farming methods and equipment used. Nonetheless, in the very few cases in which economic and social remittances have been used to intensify cultivation and introduce new crops, the investments have resulted in markedly improved returns. Since migrants from the households in this study do not typically work in agricultural employment abroad, social remittances such as the introduction of new techniques or crops are limited (see also Jones 2011).

As shown in several previous studies, agriculture has generally become a secondary livelihood as the households of López Rancho and Molino Blanco have gained access to other income sources through off-farm/non-farm employment and labor migration (see also Black 1993; McCarthy et al. 2009; Preston et al. 1997). Nevertheless, the agricultural activities are mostly maintained in families, as farming is considered a culturally important livelihood that provides subsistence and additional income when surplus products are sold (cf. Jokisch 2002; Li and Tonts 2014). It is not only those of the elderly generation who aspire to sustain farming; many of their children, including migrant children, also have plans to continue with cultivation, at least as a part-time economic activity. Moreover, community residents attempt to protect the extant agricultural land use through local policies limiting internal in-migration and land sales, as it is important from the food security perspective to have farmlands close to urban centers (see also Lerner and Eakin 2011; Moreno et al. 2016; Tacoli 2003). These efforts to maintain agricultural production can also be linked to the Andean “*ayllu* organization … built upon a foundation of kinship and reciprocity, communal land tenure, and ecological complementarity” (Beaule 2016, p. 605) and giving priority to the original community members (Pape 2009). I argue that agricultural households’ sporadic use of remittances as agricultural inputs actually counteracts urbanization. By having access to remittances, migrant families can maintain their farming practices for subsistence and occasional sales instead of being economically forced to sell their land for housing purposes. However, some families might keep their agricultural fields only for the time being and sell them later when prices are higher.

Unlike previous studies, this investigation does not identify any clear patterns of community-specific migration-induced farming practices depending on the agricultural community’s suitability for agricultural development (cf. Aguilar-Støen et al. 2016; Taylor et al. 2006). Hence, even though farming conditions are more favorable in Molino Blanco due to its plentiful irrigation water supply, efficient local governance, and self-protective policies against in-migration, its migrant households spend remittances on agricultural inputs at the same moderate level as do households in López Rancho. I argue that in general, the studied migrant households’ willingness to invest remittances in agricultural intensification is limited due to the communities’ peri-urban location. Given the communities’ proximity to the major cities, there are other opportunities for investment in comparison to rural communities located far away from the urban labor market and thus, investment in agriculture is a less relevant option. Ultimately, this investigation of Sacaban peri-urban communities provides an empirical example showing that transnational migration has not resulted in a wide agricultural change toward cash-intensive production or in a complete landscape change, known as the “new Bolivia rurality”, as has occurred in the more rural Cochabamban communities of Valle Alto (Yarnall and Price 2010, pp. 108, 117).

In conclusion, the results of this investigation are consistent with studies that show weak and mixed impacts of transnational labor migration on agriculture (cf. Gray 2009; see also Jokisch 2002). This study clearly shows that transnational migration need not result in the abandonment of agricultural activities in a peri-urban space, even if the space is already being pressured by rapid urbanization and internal in-migration (cf. Jokisch 2002; Yarnall and Price 2010). The migration-induced labor loss is compensated for through labor-saving adaptive activities, and remittances are occasionally used for agricultural inputs. On very rare occasions, households with agronomic knowledge have been able to substantially improve their agricultural production as a result of migration, indicating that technical guidance for agricultural families with access to remittances could improve their productivity. In general, however, the migrant households strive to maintain their farming practices for subsistence and as a part-time economic activity in addition to other income sources. Thereby, transnational migration and remittances function to preserve landesque-capital landscapes in the studied communities (see also Fisher and Feinman 2005; Zimmerer 1993) and to provide food security for the peri-urban households. Yet, with the exception of the very few previously mentioned examples of intensification, major investments in agricultural land are not attractive due to other opportunities provided by the communities’ proximity to the main cities. Overall, this study suggests that the existing conceptualization of studies on migration-driven agricultural change should be nuanced when applied to hybrid peri-urban spaces that are increasingly being observed even in other parts of Latin America.

## Footnotes

1 In the analysis material of López Rancho, I have also included five interviews conducted in the neighboring community of Lava Lava Alta. López Rancho was previously included in Lava Lava Alta. These communities are similar concerning their access to irrigation water, water and land governance, urbanization through in-migration, and socioeconomic conditions, but they are geographically separated by a river. Several López Rancho residents also have agricultural fields in Lava Lava Alta.

2 While the study results are based on all the interviews, quotations from certain interviews are used to illustrate specific points of interest. For the quotations presented, the interviewees were given pseudonyms to protect their privacy.
